# Neurophysiological, imaging and neurobiological markers of central fatigue in multiple sclerosis

**DOI:** 10.1093/braincomms/fcag134

**Published:** 2026-04-16

**Authors:** Alberto Benelli, Elisa Tatti, Rosa Cortese, Elisa Massucco, Ludovico Luchetti, Marco Battaglini, Javier Cudeiro, Anna de Mauro, Jian Zhang, Domenico Plantone, Patrizio Pasqualetti, Delia Righi, Francesco Neri, Maria Laura Stromillo, Alessandra Cinti, Alessandro Giannotta, Francesco Lomi, Adriano Scoccia, Giuseppe Lai, Nicola De Stefano, Monica Ulivelli, Simone Rossi

**Affiliations:** Siena Brain Investigation & Neuromodulation Lab (Si-BIN Lab), Department of Medicine, Surgery and Neuroscience, University of Siena, Siena, 53100, Italy; Department of Molecular, Cellular and Biomedical Sciences, City University of NewYork, School of Medicine, New York, NY, 10031, United States; UOC Neurologia, Department of Medicine, Surgery and Neuroscience, University of Siena, Siena, 53100, Italy; Siena Brain Investigation & Neuromodulation Lab (Si-BIN Lab), Department of Medicine, Surgery and Neuroscience, University of Siena, Siena, 53100, Italy; UOC Neurologia, Department of Medicine, Surgery and Neuroscience, University of Siena, Siena, 53100, Italy; Siena Imaging SRL, Siena, 53100, Italy; UOC Neurologia, Department of Medicine, Surgery and Neuroscience, University of Siena, Siena, 53100, Italy; Siena Imaging SRL, Siena, 53100, Italy; Department of Physiotherapy, Medicine, and Biomedical Sciences, NEUROcom (Neuroscience and Motor Control Group), CICA (Interdisciplinary Center for Chemistry and Biology), and Galician Brain Stimulation Centre, Universidade da Coruña, A Coruña, 15001, Spain; UOC Neurologia, Department of Medicine, Surgery and Neuroscience, University of Siena, Siena, 53100, Italy; The First Affiliated Hospital, Guangxi Medical University, Nanning, 530000, China; UOC Neurologia, Department of Medicine, Surgery and Neuroscience, University of Siena, Siena, 53100, Italy; Health Statistics, University La Sapienza, Roma, 00185, Italy; UOC Neurologia, Department of Medicine, Surgery and Neuroscience, University of Siena, Siena, 53100, Italy; Siena Brain Investigation & Neuromodulation Lab (Si-BIN Lab), Department of Medicine, Surgery and Neuroscience, University of Siena, Siena, 53100, Italy; Oto-Neuro-Tech Conjoined Lab, Policlinico Le Scotte, University of Siena, Siena, 53100, Italy; UOC Neurologia, Department of Medicine, Surgery and Neuroscience, University of Siena, Siena, 53100, Italy; Siena Brain Investigation & Neuromodulation Lab (Si-BIN Lab), Department of Medicine, Surgery and Neuroscience, University of Siena, Siena, 53100, Italy; Siena Brain Investigation & Neuromodulation Lab (Si-BIN Lab), Department of Medicine, Surgery and Neuroscience, University of Siena, Siena, 53100, Italy; Siena Brain Investigation & Neuromodulation Lab (Si-BIN Lab), Department of Medicine, Surgery and Neuroscience, University of Siena, Siena, 53100, Italy; Siena Brain Investigation & Neuromodulation Lab (Si-BIN Lab), Department of Medicine, Surgery and Neuroscience, University of Siena, Siena, 53100, Italy; Goldsmiths, UK Department of Psychology, University of London, London, WC1E 7HU, UK; UOC Neurologia, Department of Medicine, Surgery and Neuroscience, University of Siena, Siena, 53100, Italy; UOC Neurologia, Department of Medicine, Surgery and Neuroscience, University of Siena, Siena, 53100, Italy; Siena Brain Investigation & Neuromodulation Lab (Si-BIN Lab), Department of Medicine, Surgery and Neuroscience, University of Siena, Siena, 53100, Italy; UOC Neurologia, Department of Medicine, Surgery and Neuroscience, University of Siena, Siena, 53100, Italy; Oto-Neuro-Tech Conjoined Lab, Policlinico Le Scotte, University of Siena, Siena, 53100, Italy

**Keywords:** multiple sclerosis, central fatigue, neurophysiology, structural connectivity, functional connectivity

## Abstract

Central fatigue affects 80% of patients with multiple sclerosis, with 60% of them claiming it as the most disabling symptom. Current research often independently explores neurophysiological, structural, or functional imaging and biological underpinnings of fatigue, thus lacking a multidimensional perspective. Here, we used a multidimensional approach to investigate the functional, structural and biological underpinnings of fatigue in MS and to assess the relative contribution of each factor. A cross-sectional study was conducted with 41 patients with relapsing–remitting multiple sclerosis and 21 healthy controls (female 14) (HC). MS patients were recruited by including only those with an Expanded Disability Status Scale score < 4, and were categorized as fatigued (MS-F: 19, Female 13, FSS ≥ 4) or non-fatigued (MS-NF: 22, Female 11, FSS < 4). Over five phases, participants underwent Transcranial Magnetic Stimulation, resting-state Electroencephalography, structural and functional Magnetic Resonance, clinical assessments, and blood tests for neurofilament light chain, serum glial fibrillary acidic protein and cytokine levels. Data were analysed using both non-parametric and parametric tests, based on the data distribution. Finally, a decision-tree model was applied to predict patient group assignment. Neurophysiologically, the two patient groups differed in several domains. Those with fatigue had increased θ-band EEG power in frontocentral regions with eyes open. Transcranial Magnetic Stimulation findings indicated significantly lower intracortical facilitation in the MS-F group. Neuroimaging revealed stronger functional connectivity between nodes of the Default Mode Network, between the left temporal node and the right prefrontal node, in the MS-F group. Furthermore, fractional anisotropy via Diffusion Tensor Imaging showed reduced white matter integrity in the corticospinal tracts and corpus callosum in these patients. No significant differences were observed in lesion load, brain volumes, clinical/psychological measures, or blood sample findings linked with neurodegeneration or inflammation; the only psychological variable that differed between the two groups was the depression scale score, with MS-F patients reporting higher scores than MS-NF patients. The decision tree analysis identified both ICF and significantly lower fractional anisotropy values as the most accurate predictors of fatigue, with a classification accuracy of 84.2%. Results highlight the importance of a multidisciplinary approach in defining central fatigue in multiple sclerosis, which would emerge through subtle, subclinical, regional abnormalities of myelin integrity and clearly manifest neurophysiological evidence of impaired glutamatergic activity in motor areas. They also suggest possible biomarkers for the diagnosis of fatigue, possibly useful for eventual targeting novel neuromodulatory treatments.

## Teaser

Benelli A., Elisa T., *et al*., in their work, highlighted the importance of a multidisciplinary approach in defining central fatigue biomarkers in multiple sclerosis. Main results showed reduced myelin and impaired glutamatergic activity in motor areas. This study could be useful for the possible identification of new neuromodulatory treatments.

## Graphical Abstract

**Graphical Abstract:**
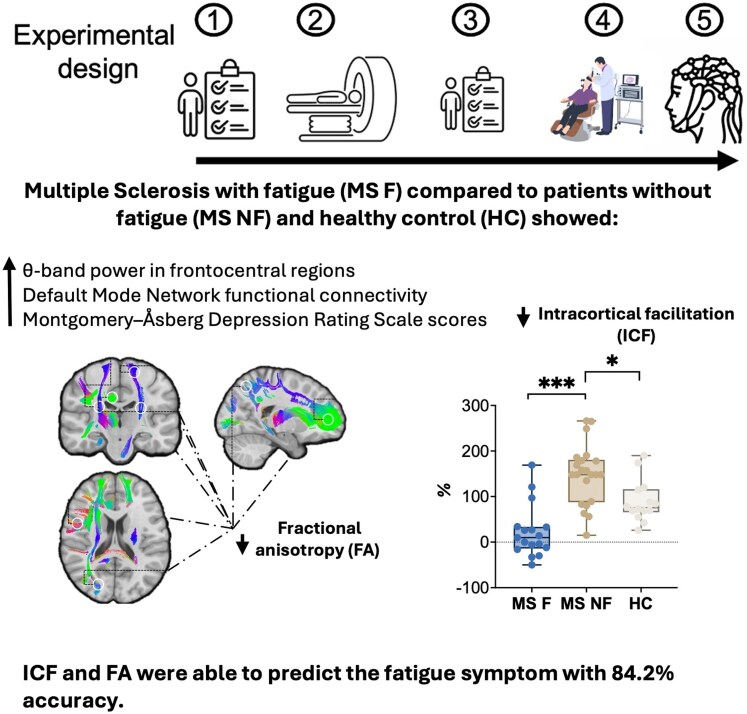

## Introduction

Multiple sclerosis (MS) is a chronic, inflammatory, and neurodegenerative central nervous system disease, commonly occurring between 20 and 40 years of age, with a higher prevalence in women.^1^ In addition to motor and cognitive impairments, 60% of patients with MS report fatigue as the most disabling symptom, significantly reducing their quality of life.^2^ Despite its prevalence, MS-related fatigue lacks a universal definition. The MS Council has defined MS chronic fatigue as ‘present for any amount of time on 50% of days for more than 6 weeks, that limits functional activities or quality of life’. Recently, a new definition has been introduced, characterizing it as ‘a chronic perception of effort and diminished resistance to sustained physical and mental activities that do not improve with rest’.^3^ This subjective symptom is assessed using self-report measures such as the Fatigue Severity Scale (FSS) and the Modified Fatigue Impact Scale.^2^

Since the disease itself is characterized by a high state of inflammation, the fatigue symptom also correlates with this. In fact, in the early stages of the disease, fatigue is associated with elevated levels of inflammatory cytokines, such as interleukin-6 (IL-6), tumour necrosis factor-α and interferon-γ (IFNγ), but not interleukin-10 (IL-10).^4-7^ These findings suggest that anti-inflammatory treatments may be most effective during this early phase. The scientific and research community interested in understanding the main characteristics of this symptom is currently debating whether it should be considered a functional or structural phenomenon. The answer remains unclear, as numerous studies over the years have provided evidence supporting both perspectives. Neuroimaging and electrophysiological techniques, in particular, have been the main tools used to investigate the symptom, revealing the coexistence of both functional and structural components.

From a structural perspective, the putative underlying mechanisms of MS-related fatigue involve both inflammatory and structural brain changes. At later stages, structural atrophy in regions such as the insula, anterior cingulate and hypothalamus becomes more prominent, possibly contributing to fatigue.^8^ Neuroimaging studies have identified correlations between fatigue and atrophy in cortical regions like the frontal and parietal lobes, as well as deep structures such as the thalamus, striatum and basal ganglia.^9,10^ Additionally, advanced imaging techniques reveal demyelination and axonal loss in the thalamus, further highlighting the role of structural damage in MS Fatigue.^11^

From a functional perspective, several studies link MS Fatigue to reduced glucose metabolism in the prefrontal, premotor and supplementary motor area (SMA), as well as in the putamen^12^; both glutamatergic and dopaminergic systems strongly influence these areas. Dysregulation of these systems, particularly cortico-striatal pathways, is a key factor in the development of MS Fatigue, as it affects reward processing and motivation.^12^ In addition, serotonergic and noradrenergic systems, which originate in the raphe nuclei and locus coeruleus, may contribute, as these systems are also implicated in depression, a common confounder of MS Fatigue.^12^

Resting state functional connectivity (rsFC) changes play a critical role in MS-related fatigue, often surpassing the weight of structural damage. Studies have reported increased activation in sensory motor networks, including the SMA and primary motor cortex (M1), along with decreased activation in the basal ganglia, essential for motivation and reward processing.^13^ Fatigue also impacts non-motor networks, such as the default mode network (DMN), with MS patients experiencing increased resting-state connectivity in the posterior cingulate cortex and decreased connectivity in the anterior cingulate cortex.^14^ These findings that functional reorganization compensates for deficits in activation.

Brain activation patterns during cognitive tasks reveal striking differences between MS patients and healthy individuals. In healthy people, mental fatigue prompts increased activation in frontal brain regions, facilitating faster cognitive processing. Conversely, MS patients display greater activation in posterior brain regions (such as precuneus, lingual gyrus and middle occipital gyrus) without corresponding improvements in processing speed.^15^ Fatigue is also linked to impaired interhemispheric connectivity between sensorimotor areas and reduced communication between the primary somatosensory cortex (S1) and M1.^16^ Additionally, MS-Fatigued patients exhibit heightened activation in regions involved in motor planning and adaptation, such as the putamen, dorsolateral prefrontal cortex and premotor cortex.^17,18^

The functional MS Fatigue hallmark, as proposed by Chaudhuri & Behan,^19^ is a ‘failure to integrate limbic inputs and non-motor functions within the basal ganglia, involving the striatal-thalamic-frontal cortical system’. This hypothesis has been corroborated by several studies in MS populations^20^ and other neurological conditions.^21^ Advanced magnetic resonance imaging (MRI) techniques have further supported the link between central fatigue, both physical and cognitive and disturbances in the cortico-striatal-thalamo-cortical (CSTC) circuit.^20^ These findings underscore the complex interplay of functional reorganization and circuit-level disruptions underlying MS-related fatigue, emphasizing the need for targeted interventions to address these mechanisms. Electrophysiological studies provide additional insights into the neurophysiological basis of MS-related fatigue. EEG has shown that fatigue correlates with altered connectivity in the parietal region and increased fronto-frontal connectivity in the β and θ bands.^22^ Additionally, transcranial magnetic stimulation (TMS) studies suggest that fatigue is associated with prolonged cortical silent periods, indicative of increased GABAergic inhibition.^23^ Decreased short-interval intracortical inhibition has also been observed, reflecting imbalances in intracortical GABAergic and glutamatergic activity.^24-26^ The frequent concomitance of depressive symptoms further complicates the interpretation and detection of specific correlates of MS Fatigue. Both depression and fatigue conditions exhibit overlapping symptoms, including reduced motivation and diminished positive affect, which stem from deficits in reward processing. This frequent co-occurrence, even during the early stages of MS, makes it difficult to identify the factors that uniquely define fatigue.^27^

Overall, MS Fatigue results from complex interactions among neuroinflammation, structural brain changes and functional reorganization within motor and non-motor networks. Current definitions primarily rely on subjective reports, leading to inconsistencies and potential misrepresentation of the symptom. This lack of an objective definition hinders effective treatment approaches.^28^

Due to the complex pathogenetic mechanisms of fatigue, it is crucial to employ multidisciplinary approaches that combine neuroimaging, biological and neurophysiological investigations, as well as cognitive assessments. Unlike previous investigations, which has typically examined only one component of this complex symptom, the current multidisciplinary study has two main goals: first, to determine whether the symptom is mainly driven by functional dysfunctions, structural alterations or both, and second, to identify which features are most predictive of fatigue status. To achieve this, participants underwent structural and functional MRI scans, EEG at rest and during cognitive and motor tasks, TMS-based neurophysiological evaluations and blood sample analysis to assess neurodegeneration and inflammation markers. Furthermore, participants completed both psychological and neuropsychological assessments, which included an evaluation of major cognitive functions as well as underlying mood disorders.

## Materials and methods

### Participants

In this cross-sectional study, we recruited 62 participants: 41 with relapsing–remitting MS and 21 healthy subjects [14 female; mean (± SD) age: 41.8 ± 10.8 years]. All patients were fully right-handed according to Oldfield’s questionnaire,^29^ and MS Diagnosis was made according to McDonald’s criteria.^30^ Based on FSS^31^ scores, patients were classified as fatigued [MS-F: 19, FSS ≥4; 14 female; mean age: 41.8 ± 10.8 years; range 24–62 years; Expanded Disability Status Scale^32^ (EDSS): median = 2.0 (min 0–max 2.5); disease duration: 107.7 ± 90.7 months] or non-fatigued [MS-NF: 22, FSS <4; 11 female; mean age: 42.4 ± 9.6 years; range 26–57 years; EDSS: median = 1.5 (min 1 to max 2.5); disease duration: 107.5 ± 91.2 months] (see Table 1 for clinical and demographic features).

**Table 1:** Clinical and demographic features

	Sample	Age	Gender	BMI	Education (years)	Disease duration (months)	FSS	EDSS
MS-F (F)	19	41.8 ± 10.8	F = 13 M = 6	24.8 ± 5.8	13.3 ± 3.5	107.7 ± 90.7	5.2 ± 0.8	1.7 ± 0.6
MS-NF	22	42.4 ± 9.6	F = 11 M = 11	25.4 ± 5.3	14.1 ± 3.1	107.5 ± 90.9	2.4 ± 0.8	1.4 ± 0.4
HC	21	41.7 ± 10.7	F = 14 M = 7	22.1 ± 2.8	16.4 ± 2.8			

Descriptive statistics and group comparisons of demographic and clinical measures for the MS-F, MS-NF and healthy control (HC) groups. BMI, body mass index; Dur, disease duration (months); Ed, education; EDSS, Expanded Disability Status Scale; F, females; FSS, Fatigue Severity Scale; HC, healthy control; M, males; MS-F, multiple sclerosis with fatigue; MS-NF, multiple sclerosis without fatigue; Samp, sample size.

The study protocol was approved by the local Ethics Committee (Brainsight 20–24 protocol), and all subjects gave their informed consent to participate. Participants underwent a five-phase investigation consisting of transcranial magnetic stimulation (TMS), resting state EEG, structural (FLAIR, T1, DTI) MRI, and resting state-functional MRI (fMRI), clinical assessment, blood test for the detection of Neurofilament light chain (NfL) and glial fibrillary acidic protein (GFAP), as well as cytokine levels detection. Exclusion criteria were contraindications for TMS (e.g. epilepsy, heart pacemaker, metallic implants in the brain, pregnancy),^33^ EDSS ≥4, relapses in the previous 3 months, paresis of the upper limb and occurrence of new neurological symptoms within 4 weeks before the investigations.

Concurrent therapies for MS, reported in the Supplementary material (Supplementary Table 1), were not interrupted.

For personal reasons, some participants did not undergo all investigations. Therefore, the final data collection and analysis covered blood samples (17 MS-F, 18 MS-NF and 12 HC), MRI/fMRI (19 MS-F, 22 MS-NF and 21 HC), TMS (19 MS-F, 22 MS-NF and 17 HC) and EEG (19 MS-F, 22 MS-NF and 18 HC).

### Experimental design

**Figure 1:**
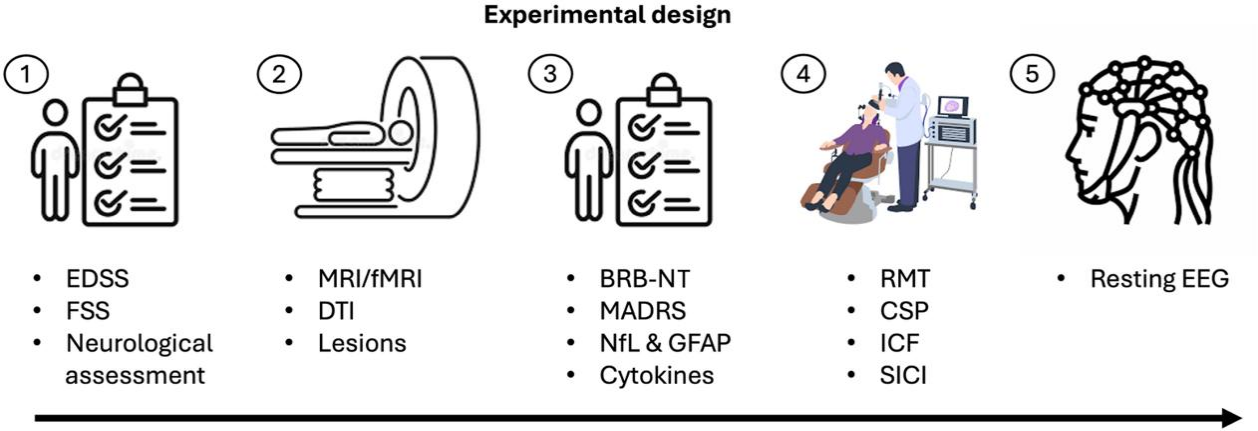
**Experimental design and summary of multidimensional investigations:** the study consists of five distinct phases (over 3 days), progressing from left to right: the first phase involves recruitment along with neurological and clinical assessment; the second phase focuses on neuroimaging investigations; the third phase centres on the collection of biological samples as well as cognitive and psychological evaluations; and the fourth phase is dedicated to gathering neurophysiological parameters. Brief Repeatable Battery of Neuropsychological (BRB-N).

Figure 1 shows the experimental setting and design. The study consisted of five phases: the first phase included clinical assessments; the second phase included MRI scans (50 min long); the third phase included both neuropsychological and psychological assessment (60 min long), and blood samples; the fourth phase neurophysiological investigations as transcranial magnetic stimulation (30 min long) and, finally, a rest EEG recording (3 min EC/3 min EO). This phase also includes task EEG recordings during a motor task, whose results are presented elsewhere,^34^ and a working-memory task.

All five assessments were performed on four different days, with a 1-day interval in between, except the TMS and EEG recording, which were performed on the same day, 2 h apart: stimulation first and then EEG; given that the single and paired pulse TMS protocols used here do not produce neuromodulatory after effects as repetitive TMS, any effect on the following EEG measurement is ruled out.

### Clinical, neuropsychological, psychological and biological assessments

All participants underwent clinical assessment with neurological examination, EDSS and FSS. The FSS is a questionnaire that contains nine statements that rate the severity of subjective fatigue symptoms. The patient must read each statement and circle a number from 1 to 7, based on how accurately it reflects their condition during the past week. Then, the neuropsychological assessment was performed using the Rao Brief Repeatable Battery^34^ to evaluate cognitive function, as well as tests for dexterity and mobility, including the 9-Hole Peg Test and the Timed 25-Foot Walk Test. The Rao Brief Repeatable Battery focuses on the cognitive domains most affected in MS. It includes tests for verbal learning and memory, such as the Selective Reminding Test (SRT), which assesses Long-term storage, consistent long-term retrieval and delayed recall. It also evaluates visual and spatial learning and memory through the 10/36 Spatial Recall Test and its delayed recall. Additionally, the battery measures complex attention and information processing speed using the Paced Auditory Serial Addition Test and the Symbol Digit Modalities Test, as well as verbal fluency based on semantic stimulus through the Word List Generation task. Depression was assessed using the Montgomery–Åsberg Depression Rating Scale (MADRS), which ranges from 0 to 60, with higher scores indicating greater severity of depressive symptoms.^35^

### Blood samples

To investigate levels of inflammation and potential parameters of neurodegeneration, blood serum samples were collected between 8 and 10 a.m. following an overnight fast. The blood was immediately centrifuged to separate the serum, which was then collected into sterile polypropylene tubes and stored at −80 °C. Through these samples, we evaluated:

### NfL and GFAP assay

The concentrations of NfL and GFAP were determined in patients’ serum samples using commercially available immunoassay kits for NfL and GFAP—SimoaTM assay Neurology 2-Plex B (GFAP, NfL) Assay Kit (Catalogue #103520; Quanterix, Billerica, MA, USA). The assays were conducted on the semi-automated ultrasensitive SR-X Biomarker Detection System (Quanterix). Samples were diluted at a ratio of 1:4 and randomly distributed on 96-well plates. Quality control samples, provided with the kit, exhibited concentrations within the predefined range, and the coefficient of variance across the plates was maintained below 10%. All samples were analysed blindly under α-numeric codes, and diagnostic codes were disclosed only after quality control–verified NfL and GFAP concentrations were reported to the database manager. Concentrations were measured in pg/ml and documented in the database.

### Cytokines assay

Concentrations of cytokines in patient serum sample were determined using a multiplex bead-based flow cytometry assay (LEGENDplex HU Essential Immune Response Panel, BioLegend, San Diego, CA, USA), which include detection of interleukin-2, interleukin-4, C-X-C Motif Chemokine Ligand 10 CXCL10 (IP-10), interleukin-1 beta (IL-1β), tumour necrosis factor-α, C-C Motif Chemokine Ligand 2 (monocyte chemoattractant protein-1) CCL2 (MCP-1), interleukin-17A (IL-17A), interleukin-6 (IL-6), interleukin-10 (IL-10), IFN-γ, interleukin-12 p70 Heterodimer (IL-12p70), C-X-C Motif Chemokine Ligand 8 (interleukin-8) CXCL8 (IL-8), transforming growth factor beta 1 TGF-β1 (Free Active Form). The FACSCanto II flow cytometer and LEGENDplex™ version 8.0 software (BioLegend, San Diego, CA, USA) were utilized for sample analysis. Samples were diluted at a ratio of 1:2 and randomly distributed on the plates. Concentrations were measured in pg/ml and recorded in the database. Before analysis, the cytometer underwent calibration using set-up beads according to the manufacturer's protocol.

### MRI acquisition and processing

MRI data were acquired with an eight-channel head coil on a 3-Tesla MRI scanner (Philips Medical Systems, Best, The Netherlands). A dual-echo, turbo spin-echo sequence [repetition time (TR)/echo time (TE)1/TE2 = 4000/10/100 ms, voxel size = 1 × 1 × 3 mm] yielded proton density and T2-weighted (T2W) images. Diffusion tensor imaging (DTI) data were acquired using an echo-planar imaging sequence (TR = 7036 ms; TE = 196 ms; voxel size = 2.5 mm^3^) with 32 diffusion directions and *b*-value = 900 s/mm^2^. DTI acquired had these parameters: TR = 7036 ms; TE = 196 ms, flip angle = 90°, voxel size = 2.5 mm3, 50 slices, diffusion sampled on the half sphere with 32 diffusion directions and b-value = 900 s/mm^2^; fMRI acquired had these parameters: 200 volumes, TR = 3000 ms, TE = 35 ms, flip angle = 90°, voxel size = 1.87 × 1.87 × 4 mm, 30 slices.

A high-resolution T1-weighted image (T1W, TR = 10 ms, TE = 4 ms, voxel size = 1 mm^3^) was also acquired for image registration, anatomical mapping and analysis of grey matter (GM) concentration. All images were visually checked and analysed centrally.

Brain tissues and structures masks were created using an in-house developed pipeline, using FSL tools (https:fsl.fmrib.ox.ac.uk). To investigate voxel-wise differences in GM and White Matter (WM) volumes, we first created a study-specific GM and WM mask template. This was performed by registering each subject-specific GM and WM mask to a standard-specific space. Then, each patient-specific GM and WM mask was registered on the previously created GM and WM templates. These acquisitions were used to evaluate both structural and functional parameters.

### Brain volumetric assessment

Brain volumes were segmented using T1-weighted (T1-W) images after performing lesion filling as described by Battaglini *et al*.^36^ Following this step, brain segmentation was conducted using SIENAX2.^37^ SIENAX2 employs several FSL tools^38^ to perform image intensity correction, segmentation and parcellation of the parenchyma. The tool is designed to automatically identify and label different brain structures (for example, GM, WM, CerebroSpinalFluid, thalamus and hippocampus). Cortical GM is parcellated using the Desikan–Killiany cortical atlas.^39^ Additionally, the tool divides the brain into lobes (frontal, occipital, parietal and temporal lobes) and functional networks (sensorimotor network II, secondary visual network, DMN, executive control network, left and right working memory networks and frontoparietal attention network). Cortical thickness was estimated using FreeSurfer (https://surfer.nmr.mgh.harvard.edu/fswiki/FreeSurferAnalysisPipelineOverview).

### WM lesion detection and analysis

WM lesions were segmented semi-automatically on FLAIR/T2-hyperintense images using a custom deep-learning tool to create lesion masks. Each lesion was reviewed by two experts (R.C., and AdM) and, if needed, manually corrected using FSLview (FMRIB Software Library, https://fsl.fmrib.ox.ac.uk/fsl/docs/#/). Volume was then extracted from the manually revised lesion masks.

Each participant’s binarized lesion mask was registered to the MNI152 standard brain through FSL (FMRIB Software Library, www.fmrib.ox.ac.uk/fsl) using linear registration (FLIRT) (Jenkinson & Smith, 2001), followed by nonlinear registration (FMRIB Non-linear Image Registration Tool).^40^

Finally, a lesion probability map (LPM) was generated, first merging and then averaging all lesion masks previously registered onto the standard brain.

Finally, we observed which tracts were most affected by the lesions and whether there were differences between the groups, considering the number of lesion voxels that fell in the tract considered.

Each lesion voxel was associated with the traits in the atlas (JHU ICBM 1 mm) (https://neurovault.org/collections/264/) with different percentages.

Each lesion mask was examined to verify correspondence with the dimensions of the reference atlas. In cases of discrepancy, the masks were resampled to align with the atlas using calculated scaling factors and linear interpolation, implemented via the zoom function from the SciPy library.

### WM preprocessing and DTI analysis

DTI preprocessing was carried out using the FSL programme *Eddy*, which corrects for eddy current distortions, subject movement and signal dropout.^41^ The FMRIB Diffusion Toolbox was then used to create fractional anisotropy (FA) and mean diffusivity (MD) images by fitting a diffusion tensor model to each voxel, followed by brain extraction with BET (Smith *et al*., 2002). All FA and MD no-smoothed images were registered to a standard-space image (FMRIB58).^42^ FA and MD values were extracted by performing tract-based spatial statistics,^43^ part of the FSL suite.^38^ Tract-based spatial statistics has been used because it provides a robust, automated framework for voxel-wise analysis of white matter microstructure across subjects.

For region-based analysis, mean FA and MD voxel intensity values were calculated within specific white matter tracts defined by the JHU-ICBM (1 mm) (https://neurovault.org/collections/264/) white matter atlas.

### fMRI preprocessing and analyses

Functional and anatomical MRI data processing and statistical analyses were performed using the CONN toolbox (RRID: SCR_009550), version 17.f^44^ and SPM version 12 release 7771 (Wellcome Department of Imaging Neuroscience, UCL, London, United Kingdom) in MATLAB R2022a (The MathWorks Inc., Natick, MA, USA) using CONN's default minimal preprocessing pipeline. This pipeline includes the functional *realignment and unwarp* for intermodality coregistration of all scans to the first scan, *slice-timing correction* STC^45^; compensating for acquisition time differences among different slices, *outlier detection*^46^ identifying individual scans with suprathreshold framewise displacement and/or global signal change (GSC) values, *direct functional normalization*^47^ projecting functional images into standard Montreal Neurological Institute 152 (MNI) reference space resampled to 2 mm isotropic voxels, and spatial *smoothing* with a 8 mm full width at half maximum Gaussian kernel. Anatomical data preprocessing comprised *direct segmentation and normalization,*^48^ which iteratively performed tissue *segmentation* into six tissue classes, including GM, WM and CerebroSpinalFluid, using SPM12 posterior tissue probability maps and *normalization* to IXI-549 MNI space, resampling the output anatomical images to 2 mm isotropic voxels.

### Neurophysiological data collection and analysis

#### TMS recordings and analyses

TMS was performed with a butterfly focal 70-mm coil connected with an ATES magnetic stimulator (EBNeuro, Italy). With the participant seated, the TMS coil was maintained tangentially to the scalp with the handle pointing backward and laterally at an angle of 45° from the midline perpendicular to the central sulcus. This coil position delivers posterior-anterior directed pulses that are known to produce large motor-evoked potentials (MEPs) in contralateral hand muscles, resulting from the gradual recruitment of indirect waves.^49,50^ The cortical representations of the first dorsal interosseous were targeted within the left M1. The use of a focal coil guaranteed stable simultaneous MEPs from the considered hand muscle. To measure electromyography (EMG) activity and collect the MEPs, foam surface electrodes were placed on the belly of the first dorsal interosseous muscle, and the ground and the reference electrodes were placed on the styloid process and the interphalangeal joint of the index finger, respectively.

Once the best fitting scalp position for the coil for targeting M1 was determined, this was maintained throughout the experiment using a neuronavigation system (BrainNET, EBNeuro Ltd, Florence, Italy) using infrared cameras (Polaris Vicra, NDI, Waterloo, Canada). An MRI template for each participant was uploaded in the neuronavigation software, and a co-registration procedure was performed using scalp landmarks (nasion, vertex and the two preauricular points) and additional landmarks positioned on a plastic glasses frame worn by the subjects. The coil was calibrated using an in-house algorithm based on five landmarks specific to the device. During TMS, the software provided online visual feedback, allowing the investigators to keep constant (with a tolerance of 2 mm for each spatial *x*, *y* and *z*-axis) the desired coil orientation/rotation/distance during the whole session.

Corticospinal responses (or MEPs) were recorded using surface electrodes placed with a belly/negative-tendon/positive montage; the analysis time base was 50–100 ms, bandpass acquisition filters were 20–20 000 Hz, and the gain of amplifiers was adjusted to entirely show the negative-positive evoked peaks of MEPs, without saturation.

Resting motor thresholds were determined as the minimum amount of intensity of the TMS, regarding the maximal stimulator output (0–100%), necessary to elicit 5 out of 10 MEPs with a peak-to-peak amplitude of 50 μV during muscle relaxation.^51^ To measure CSP, 15 pulses at 120% of RMT were delivered,^51^ while the subject produced 20% of his maximal contraction during a pinch grip between his index finger and thumb.^52^ The EMG signal was continuously displayed to both the subject and the experimenter and served as a feedback mechanism to maintain the same level of contraction throughout the CSP measurement. For all subjects, CSP was measured as the length of time from the MEPs’ peak (+2SD from baseline EMG mean) to the onset of the return of voluntary tonic EMG activity. The duration of CSP was obtained by averaging 10–15 traces for each subject.^51^ MEP onset was determined as the time point where the MEP exceeded ±2SD from the EMG background activity.^51^

Paired TMS was applied to test short intracortical inhibition (SICI) and intracortical facilitation (ICF).^24^ The first subthreshold stimulus was delivered with 80% of MT at rest, the second, test stimulus was set at 120% of the MT at rest, producing a MEP of ∼1 mV. For SICI, a 3-ms interstimulus interval (ISI) and ICF, a 10-ms ISI, was chosen. For each ISI, 15 stimuli were given randomly, 5–8 s apart from each other. Various studies have demonstrated that this paired-pulse paradigm tests the excitability of intracortical GABA-A-ergic (SICI) and glutamatergic (ICF) neuronal circuits.^52-54^ Recordings were made during complete muscle relaxation. All subjects’ recordings were taken from the right first dorsal interosseous muscle with surface electrodes employing biphasic stimuli for RMT, CSP, SICI and ICF. Results were stored on an EMG machine (Next, EB Neuro) and analysed offline.

The RMT values were exported directly from the stimulator, whereas for the CSP, a GABA-B-ergic event,^51^ the length between the peak of the MEP and the first observed continuous muscle activation was measured. For the paired pulse protocols, such as ICF and SICI, to compute the final index used in the subsequent analyses, we calculated the average amplitude of all unconditioned (test) MEPs and the average amplitude of all conditioned MEPs for each interstimulus interval.

#### EEG preprocessing and analyses

EEG recordings were acquired at rest using a 64-channel cap with a G-Tech amplifier system (g.®HIamp). Electrode placement followed the international 10–20 system, with two additional electrooculography electrodes positioned vertically above and below the left eye and two electrodes on the left and right mastoids integrated into the cap. The sampling rate was 256 Hz, and the reference electrode was placed on the nasion (Nz). Electrode impedances were maintained below 10 KΩ throughout the recording. Data preprocessing was performed using the open-source EEGLAB toolbox^55^ for MATLAB (Mathworks, Inc.).

The continuous signal was first high-pass and low-pass filtered between 1 and 90 Hz using a finite impulse response filter and Notch filtered at 50 Hz (46–54 Hz) to remove line noise. Filtered data were visually inspected to remove channels with bad signal quality. To remove stereotypical artefacts such as eye blinks, muscular activity and heartbeat, we ran independent component analysis using the infomax algorithm and artefactual components were automatically rejected using the ICLabel Toolbox for EEGLAB. Removed scalp electrodes were reconstructed using spherical spline interpolation. External channels were also removed, resulting in 61 averaged and referenced channels.

Power spectral analysis was carried out using the MATLAB-based Fieldtrip Toolbox.^56^ Power spectra across the 1–80 Hz frequency range (0.5 Hz steps) was computed using a Hanning taper, minimizing spectral leakage and reducing estimation variance. Spectral activity for the significant electrodes (see statistical analyses below) was extracted to compute additional analyses to link EEG findings with the other measures.

### Statistical analyses

Regarding statistical analysis on extracted data, we used IBM SPSS Statistics 29 software by performing a Shapiro-Wilk test for normality check; then, we used the Kruskal–Wallis H test for the nonparametric data analyses (TMS values, lesions, DTI scores, blood samples, EDSS and cognitive scores). For analysis of the parametrically distributed data (brain volumes and fMRI scores), we used ANCOVA analysis through JASP software, version 0.19.0 (JASP Team, 2024). MADRS scores were used as covariates to test differences between groups. All significant reported results are *P* < 0.05, Bonferroni corrected.

Group-level fMRI analyses were performed using a General Linear Model.^57^ For each connection, a separate General Linear Model was estimated, with first-level connectivity measures at this connection as dependent variables (one independent sample per subject and one measurement per task or experimental condition, if applicable) and groups or other subject-level identifiers as independent variables. Connection-level hypotheses were evaluated using multivariate parametric statistics with random effects across subjects and sample covariance estimation across multiple measurements. Inferences were performed at the level of individual clusters (groups of similar connections).

Cluster-level inferences were based on parametric statistics within and between each pair of ROIs,^58^ with ROIs identified using a complete-linkage hierarchical clustering procedure based on ROI-to-ROI anatomical proximity and functional similarity metrics. Results have been thresholded utilizing a combination of a *P* < 0.05 connection-level threshold and a familywise corrected p-FDR < 0.05 cluster-level threshold.^59^ Analyses were performed on the structural-functional atlas Schaefer (https://github.com/ThomasYeoLab/CBIG/blob/master/stable_projects/brain_parcellation/Schaefer2018_LocalGlobal/README.md) to examine the primary differences among key functional networks, including the DMN and SMN. Group comparisons have been performed using an ANCOVA statistic because the data followed a normal distribution, verified via the Shapiro–Wilk normality test.

To evaluate differences in GM atrophy and lesion load, means, medians, proportions of demographics and clinical features were calculated for patients (and their subgroups) and healthy controls. Differences were evaluated using ANOVA. Differences in GM volumes and lesion load between each patient group and the control group, and between patient groups, were assessed using design matrices within general linear models with disease as the variable of interest and age and sex as covariates. The voxel-wise analyses used the GM and lesion masks as input images for the ‘randomize’ tool (i.e. the dependent variable). Within these masks, the randomized tool detects regions with increased GM atrophy or increased lesion load. For the significant results (*P* < 0.05, corrected for multiple comparisons), the number of voxels (nV) is reported for GM atrophy, and the number of voxels and coordinates in the MNI space of the most significantly different voxels are reported for lesion load map (LPM).

Finally, EEG group comparisons for both the resting conditions were conducted using a two-tailed independent samples *t*-test, with the false discovery rate (FDR) correction for multiple comparisons. The significance threshold was set at 0.05, and 10,000 randomizations were used for the permutation test. The analysis focused on θ (4–7 Hz), α (8–13 Hz) and β (14–25 Hz) frequency bands. Also, MADRS scores were also included as covariates in the EEG analysis, which focused on resting-state power spectra.

Outliers were detected using SPSS through Tukey’s method and then excluded from the analyses. Aiming to test if significant variables correlate with the FSS scale, we used a Spearman correlation analysis; EEG correlation analyses were performed considering the θ-band only based on the strength of existing results regarding fatigue. Finally, to understand the extent to which the significant variables were able to determine the probability of belonging to the MS-F or MS-NF group, we used a Decision Tree in MATLAB with the Classification Learner application, the Statistics and Machine Learning Toolbox (version 11.4), and as inputs, we chose all those variables that resulted significant to previous statistics and significantly correlated with FSS scores in MS-F group. For this last analysis, we considered MS patients only because the variable to be estimated was the group variable (1 = MS-F; 2 = MS-NF), without including HC.

Classification accuracy was defined as the ratio of [true positives (TP) + true negatives (TN))/(TP + TN + false positives (FP) + false negatives (FN)].

## Results

No differences were found among the three groups regarding gender (*P* = 0.193) and age (*P* = 0.969); on the other hand, education level was higher in the healthy group than in the MS-F group of patients (*P* = 0.013). No differences were found between the groups concerning BMI (*P* = 0.091) and disease duration (*P* = 0.995), but a significantly higher EDSS was found for patients with fatigue compared to patients without fatigue (*P* = 0.005), although both groups scored below 2, indicating a mild disability. Additionally, MS-F patients reported a significantly higher score at MADRS (Supplementary Fig. 1) than both the healthy control group (*P* = <0.001), indicating a ‘very mild’ to ‘mild’ depression, and MS-NF patients (*P* = 0.012) and lower scores at Multiple Sclerosis Quality of Life-54 (MSQOL-54) (*P* = 0.018). Descriptive results are reported in Table 1 as the mean [ ± SD]; for EDSS only, the median [ ± SD] is also reported. Results related to neuropsychological analysis (Supplementary Table 2), clinical blood samples (Supplementary Table 3), and both brain volumetry and atrophy (Supplementary Fig. 2) are reported in the supplementary materials, as no significant difference emerged.

### fMRI

**Figure 2:**
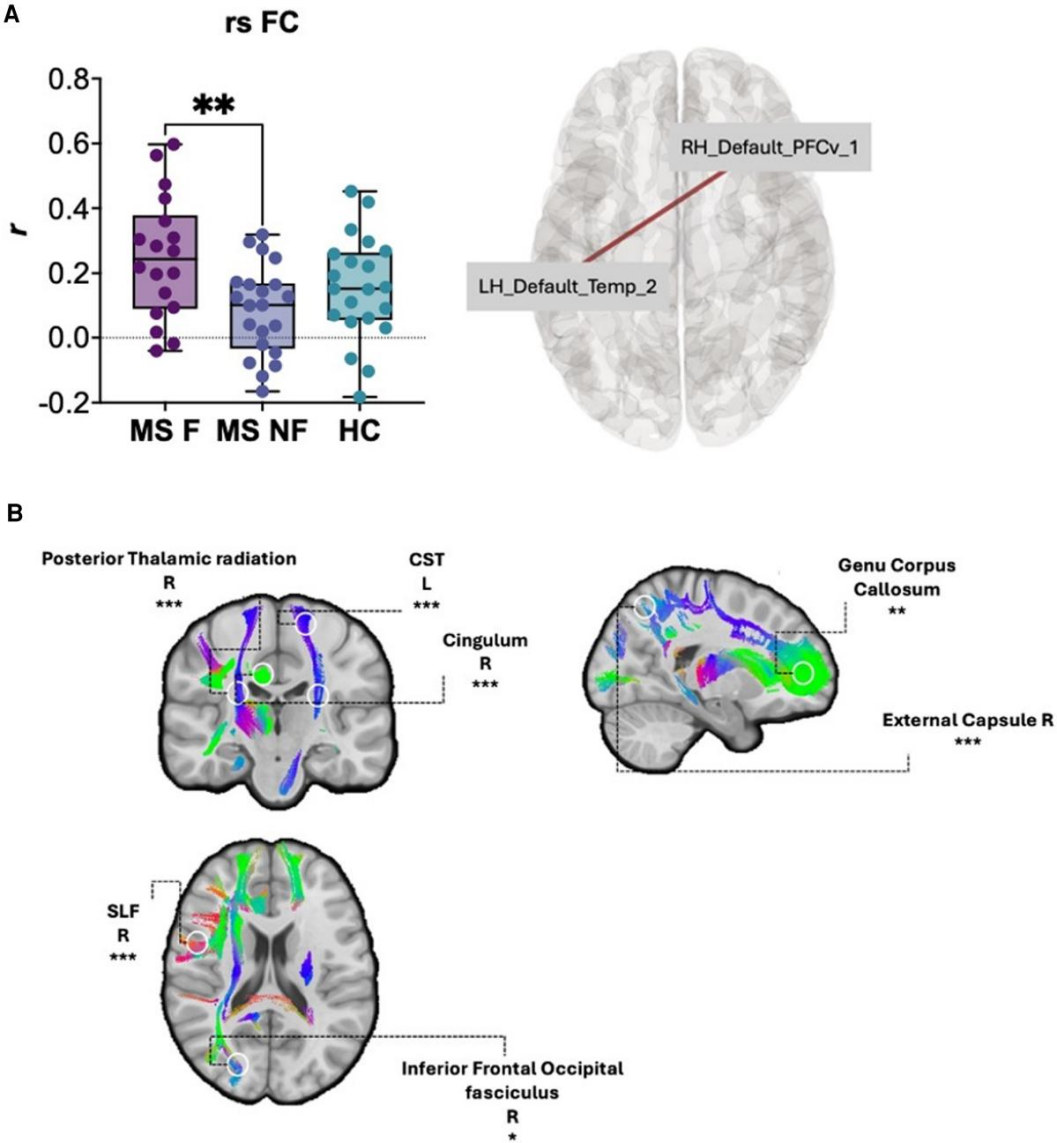
**fMRI and FA group comparisons:** (**A**) Shows correlations coefficients (r) between the left hemisphere default mode network (LH_Default_Temp_2) and the right hemisphere prefrontal node of the default mode network (RH_Default_PFCv_1), comparing the three groups; ROIs displayed represent the left hemisphere temporal node of the default mode network (LH_Default_Temp_2) and the right hemisphere prefrontal node of the default mode network (RH_Default_PFCv_1). Comparisons between multiple sclerosis patients with fatigue (MS-F), multiple sclerosis patients without fatigue (MS-NF), and healthy control subjects (HC), **P < 0.01. Each data point represents one subject. (**B**) Shows the White Matter bundles in which fractional anisotropy is reduced in the multiple sclerosis patients with fatigue (MS-F) group compared with multiple sclerosis patients without fatigue (MS-NF). The colours represent the mapping of the position and direction of the strokes: red for left-right, blue for upper-lower, and green for front-back. Data from 60 subjects were used for both analyses. For the fMRI analysis, the ANCOVA test has been used; for the FA analysis, the Kruskal–Wallis *H* test has been used. * *P* ≤ 0.05, ** *P* ≤ 0.01, ***P ≤ 0.001.

Figure 2A shows the results of the statistical analysis comparing the MS-F and MS-NF groups. The MS-F group exhibited higher rsFC between the left temporal default mode network (LH_Default_Temp_2) and the right ventral prefrontal node of the default mode network (RH_Default_PFCv_1). Comparing rsFC, the ANCOVA revealed a significant effect of GROUP [F_(2, 58)_ = 4.532, *P* = 0.015, η^2^ = 0.125] but not MADRS [F_(1, 58)_ = 1.391, *P* = 0.243, η^2^ = 0.019]. These results suggest that GROUP only contributes meaningfully to explaining variability in rsFC. *Post hoc* pairwise comparisons revealed that MS-NF had significantly higher rsFC scores compared to MS-F (mean difference = 0.198, SE = 0.067, t = 2.948, pbonf-adj = 0.014). We also compared functional connectivity among SMN nodes but found no significant differences between groups [F_(2, 58)_ = 0.506, *P* = 0.60, η^2^ = 0.017].

### Diffusion tensor imaging

This section focuses exclusively on statistically significant findings. Figure 2B highlights white matter bundles in which MS-F patients have significantly reduced FA compared with MS-NF patients. No significant differences in MD were observed between the groups. Table 2 reports FA group comparisons.

**Table 2:** FA group comparisons

	MS-F Vs MS-NF (Statistic)	CI lower	CI upper	MS-F vs HC (Statistic)	CI lower	CI upper	MS-NF vs HC (Statistic)	CI lower	CI upper	MS-F vs MS-NF (*P* values Bonf adj. corr.)	MS-F vs HC (*P* values Bonf adj. corr.)	MS-NF vs HC (*P* values Bonf adj. corr.)
Genu of corpus callosum	−18.24	−0.159	−0.030	−14.21	−0.136	−0.005	4.02	−0.039	0.087	0.038	0.004	1.000
Corticospinal tract L	−20.53	−0.154	−0.046	2.62	−0.050	0.0059	23.16	0.052	0.157	0.001	1.000	0.000
Posterior thalamic radiation R	−17.51	−0.119	−0.015	−0.218	−0.048	0.057	17.29	−0.021	0.122	0.002	0.002	0.97
External capsule R	−19.99	−0.135	−0.042	21.9	−0.041	0.052	1.91	0.049	0.139	<0.001	<0.001	0.738
Cingulum R	−17.77	0.028	0.145	−7.35	−0.097	0.021	−25.12	−0.182	−0.068	0.005	0.594	0.00
Superior longitudinal fasciculus R	−21.42	−0.132	−0.037	−2.02	−0.057	0.038	19.39	0.029	0.121	0.000	1.000	0.001

A comparative analysis of the three groups concerning significantly different white matter bundles. The Statistic output summarizes how much the group medians differ, meaning that the greater the value, the greater the difference between the groups. CI, confidence interval; HC, healthy controls; L, left; MS-F, multiple sclerosis with fatigue; MS-NF, multiple sclerosis without fatigue; R, right.

### Neurophysiology

#### Transcranial magnetic stimulation

**Figure 3:**
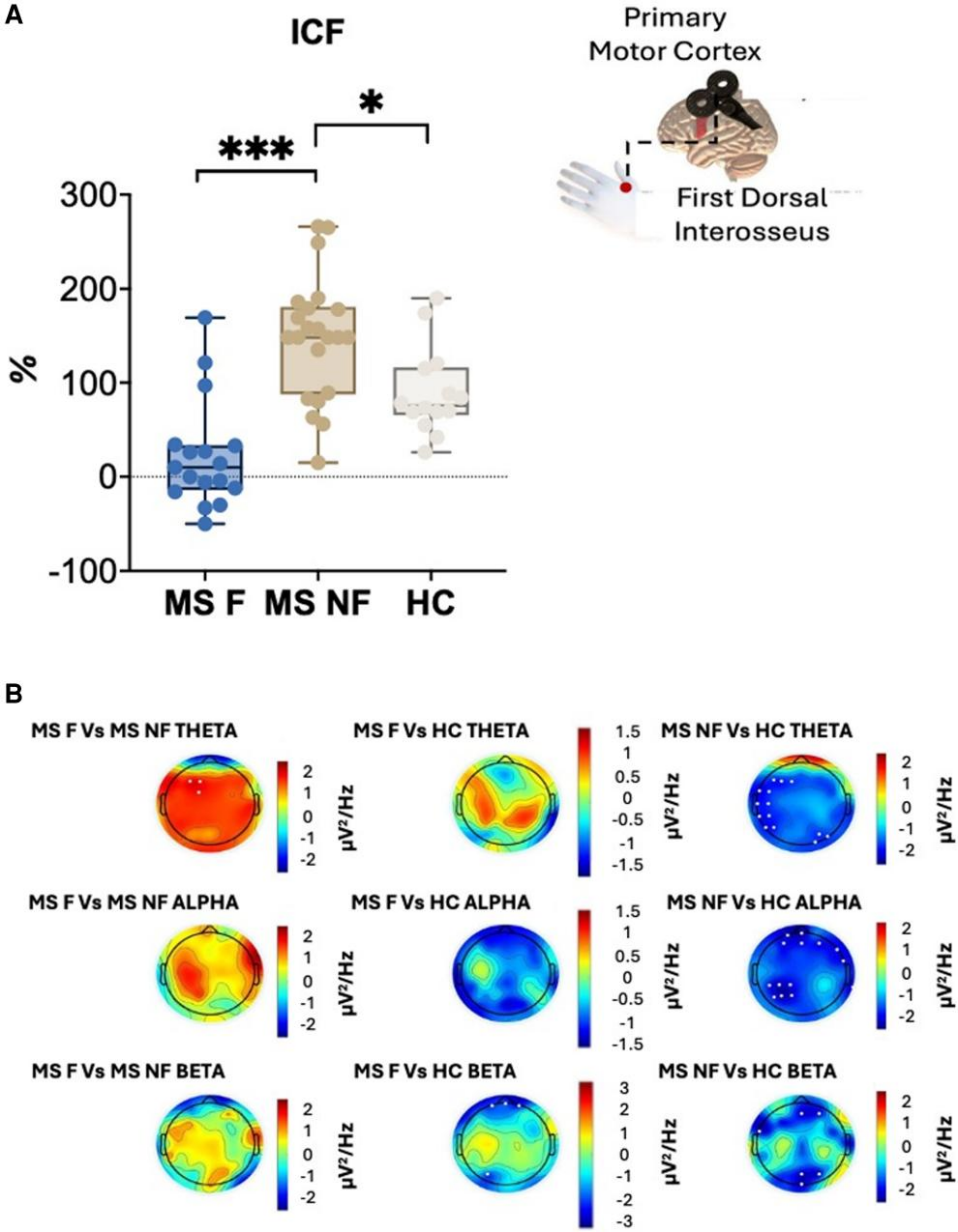
**ICF and EEG group comparisons:** (**A**) Shows the intracortical facilitation (ICF) comparisons between the 3 groups. The central line represents the median, while the whiskers at the ends correspond to the minimum and maximum values. Each data point represents one subject. The % symbol represents the change in the ICF parameter. (**B**) Reports the significant EEG differences between groups during the eyes-closed condition. All white dots reported here refer to significant electrodes. Data from 53 subjects were used for both analyses. For the ICF analysis the Kruskal–Wallis *H* test has been used; for the EEG analysis the two-tailed independent *t*-test has been used. *P < 0.05, ***P < 0.001.

The TMS parameters listed below concern the values related to RMT, SICI, CSP and ICF. For statistics, we used the MADRS score as a covariate, given the functional nature of these parameters and the possibility that mood disturbance could influence cortical excitability. Table 3 reports TMS group comparisons, and Fig. 3A shows significant ICF group differences. Regarding CSP, there was a trend (*P* = 0.085) towards shorter CSP in MS-F patients, which became significant after removing one outlier.

**Table 3:** TMS results

	MS-F vs MS-NF (statistic)	CI lower	CI upper	MS-F vs HC (statistic)	CI lower	CI upper	MS-NF vs HC (statistic)	CI lower	CI upper	MS-F vs MS-NF (*P* values Bonf adj. corr.)	MS-F vs HC (*P* values Bonf adj. corr.)	MS-NF vs HC (*P* values Bonf adj. corr.)
**RMT**	−0.76	−7.887	6.251	0.66	−2.813	11.480	1.58	−1.734	12.037	1	1	0.354
**SICI**	1.38	−1.486	33.580	0.32	−7.317	28.134	−1.7	−22.718	11.441	0.518	1	0.865
**ICF**	**−6**.**5**	−168.656	−87.525	**−3**.**01**	−110.511	−28.489	**3**.**32**	−19.076	98.106	**<**0.**001**	0.112	**0**.**005**
**CSP**	−2.76	−65.400	3.148	−2.17	−54.263	15.037	0.34	−21.873	44.900	0.081	0.103	1

Comparisons between indices extracted from the TMS across MS-F, MS-NF and HC groups. CI, confidence of interval; CSP, cortical silent period; HC, healthy control; ICF, intra cortical facilitation; L, left; MS-F, multiple sclerosis with fatigue; MS-NF, multiple sclerosis without fatigue; R, right; RMT, resting motor threshold; SICI, short intra cortical inhibition. The Statistic output summarizes how much the group medians differ, meaning that the greater the value, the greater the difference between the groups. Bold values indicate statistically significant values.

#### EEG

EEG differences between the three groups across all frequency bands were observed after FDR correction in both the eyes-open and eyes-closed conditions (*P* < 0.025 for both). Resting-state EEG analysis revealed that, during the eyes-open condition, the MS-F group exhibited higher θ band power in frontocentral regions compared to the MS-NF group (Fig. 3B). Group comparisons using the MADRS scale as a covariate are reported in the supplementary materials (Supplementary Table 4).

#### Correlation results

We conducted correlation analyses between individual FSS scores in patients with fatigue and all variables that, in previous analyses, distinguished MS patients with and without fatigue (Table 4). While all the variables considered showed a statistical difference between MS-F and MS-NF, not all of them correlate with the FSS. In fact, only the following correlate with the FSS: MADRS scores, myelin sheath integrity values, resting functional connectivity, and glutamatergic system activation. Specifically, scores on the depression scale and resting functional connectivity correlate positively, while the others correlate negatively.

**Table 4:** Correlations between FSS scores and variables distinguishing MS-F and MS-NF patients

			Spearman (ρ)	*P* value
FSS	&	MADRS	0.8	<0.0001
		ICF	−0.7	<0.0001
		Genu_CC	−0.7	<0.0001
		CST L	−0.5	0.0018
		PTR R	−0.6	<0.0001
		Ext Caps R	−0.4	0.0132
		Cing R	−0.6	<0.0001
		SLF R	−0.7	<0.0001
		Inf FoF R	−0.8	<0.0001
		rsFC	0.5	<0.001
		EO θ F1	0.07	0.652
		EO θ FC1	0.1	0.397
		EO θ F3	−0.01	0.911

Spearman correlations between Fatigue Severity Scale (FSS) scores and significant neurophysiological, imaging, and clinical variables in patients with fatigue. CC, corpus callosum; Cingulum; CST L, cortico spinal tract left; EXT_Caps, external capsule; cingulum; ICF, intracortical facilitation; inf FoF, inferior fronto-occipital fasciculus; MADRS, Montgomery-Asberg Depression Rating Scale; Post_LimC, posterior limb of internal capsule; PTR, posterior thalamic radiation; rs FC, resting state functional connectivity.; SLF, superior longitudinal fasciculus; θ, Theta.

Significant negative correlations were found between MADRS scores and ICF (ρ = −0.433, *P* = 0.005), highlighting that a higher score of depression (corresponding to a clinically mild level) is associated with reduced glutamatergic activity; similarly, MADRS scores negatively correlated with white matter tracts such as Genu (ρ = −0.323, *P* = 0.039), right posterior thalamic radiation (PTR R) (ρ = −0.441, *P* = 0.004), right Superior Longitudinal Fasciculus (SLF_R) (ρ = −0.355, *P* = 0.032) and right Inferior fronto-occipital fasciculus (Inf_FOF R) (ρ = −0.555, *P* < 0.001). When we looked at the correlation results in more detail, we also observed that ICF was positively correlated with Genu (ρ = 0.401, *P* = 0.009), Cortico Spinal Tract left (CST_L) (ρ = 0.334, *P* = 0.033), PTR R (ρ = 0.397, *P* = 0.01), Ext. cap. R (ρ = 0.316, *P* = 0.044), SLF_R (ρ = 0.634, *P* < 0.001) and Inf_FOF R (ρ = 0.38, *P* = 0.014), suggesting a link between glutamatergic activity and white matter integrity of specific tracts. No significant correlations were found between MADRS scores and any EEG *θ* power measures.

#### Classification results

**Figure 4:**
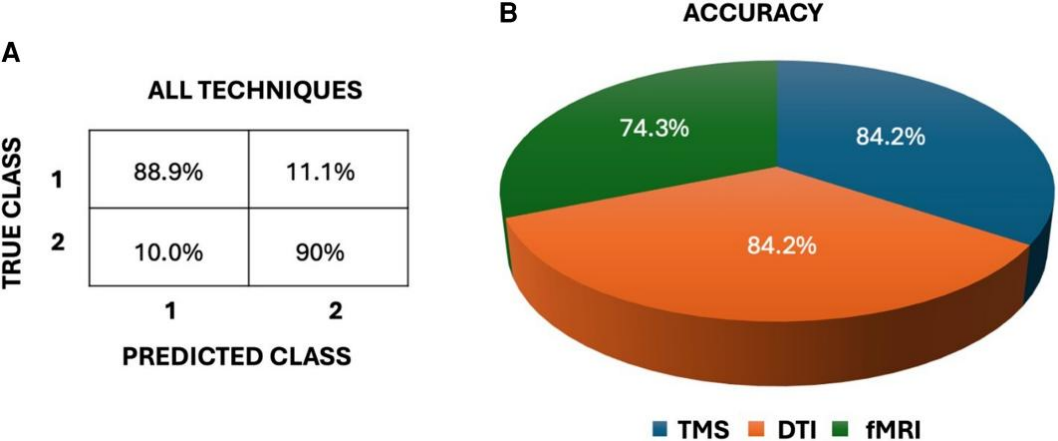
**Classification model results:** (**A**) Shows the percentage of accuracy in categorizing subjects into their appearance group: 1(MS-F) and 2(MS-NF). (**B**) Shows the percentage of accuracy in categorizing subjects in their appearance group for each technique. Data from 41 subjects were used for this analysis.

Finally, a decision tree model was implemented as a classification method to differentiate between the MS-F and MS-NF groups. Using the full set of selected variables (ICF, FA values from Genu_CC, CST L, PTR R, Ext Caps R, Cing R, SLF R, Inf FoF R, and rsFC), the model achieved an overall prediction accuracy of 89.5%, enabling a reliable distinction between the two groups. According to the inclusion criteria of the classification model, the considered variables were related to TMS, DTI and fMRI. To explore the relative contribution of each modality, we then applied the same classification approach separately to TMS, DTI, and fMRI measures. Figure 4 illustrates the contribution of each modality to group classification in relation to fatigue. In addition to the impact of key variables on symptom differentiation, the technique was most effective at distinguishing patients with fatigue from those without fatigue. Our findings revealed that both ICF and FA values, measured via TMS and DTI, demonstrated the highest accuracy, discriminating patients with 84.2% accuracy, followed by rsFC measured via fMRI with 74.3% accuracy (Fig. 4).

## Discussion

The current study addresses the still unmet need to investigate the complex, multifactorial symptom of central fatigue in MS from a multidimensional perspective, considering structural and functional neuroimaging, biological and neurophysiological aspects within the same cohort of patients.

Beyond demonstrating the feasibility of studying central fatigue through a multidisciplinary lens, our goals were to identify which parameters most powerfully drive or reflect this debilitating symptom, to identify the most discriminative parameters that differentiate MS patients with and without fatigue and, crucially, to clarify the longstanding question regarding the functional/structural dichotomy about the origin of central fatigue. In addressing this, our work departs significantly from previous research and offers new insight into the underlying mechanisms of fatigue.

It is essential to consider that MS-F patients showed higher levels of depression scores, while MS-NF patients did not differ from HC. Although the MADRS scores (Supplementary Fig. 1) indicated a mild-moderate depression (indeed, antidepressive therapies were not required in these patients), the well-established link between depression and fatigue^60^ prompted us to account for depression by including MADRS as covariate in our statistical analyses of TMS and fMRI measures (i.e. the ones more discriminating the two patients groups). Importantly, we chose not to exclude patients with mild depression to avoid stripping the study of its real-world relevance: fatigue in MS rarely occurs in isolation, and our cohort fully reflects the daily clinical reality. However, it remains difficult to distinguish whether these MADRS scores, which indicate the presence of mild depression, are related to the symptom of fatigue or to the MS diagnosis.

Beyond a slightly elevated MADRS score in MS-F patients, no other significant cognitive/psychological deficits were observed in our sample of patients (Supplementary Table 2). So, we did not confirm previous observations linking fatigue with cognitive decline.^61^

Central fatigue in MS has long resisted clear characterization because prior studies have focused on isolated domains—structural imaging, functional connectivity, immunological profiles, electrophysiology or clinical symptoms—each offering only a fragment of the full picture. We instead integrated these domains within a unified framework, analysing structural, functional, biological and neurophysiological factors in concert. This allowed us to both distinguish fatigued from non-fatigued MS patients with greater accuracy and to pinpoint which variables within the same cohort most strongly correlate with the emergence of central fatigue. By bridging disciplines and examining fatigue through multiple interconnected pathways, this study advances the understanding of one of MS’s most elusive and impactful symptoms and opens new avenues for targeted interventions.

### Inflammation and neural degeneration

Despite previous studies linking fatigue to inflammation,^8^ current findings revealed no significant differences in inflammation markers between the MS-F and MS-NF groups. This may be attributed to the concurrent anti-inflammatory and immunomodulating therapies that all patients were undergoing, which were not discontinued during the examinations to avoid clinical worsening. Still, we ensured there were no differences between the two patient groups to control for this potentially confounding factor.

Also, markers of neural degeneration were not different between patients and controls and between MS-F and MS-NF patients (Supplementary Table 3). This may be explained by similar disability levels between the two MS groups of patients, and comparable cortical and subcortical atrophy.^62^

### Structural and functional neuroimaging

We also explored structural and functional brain changes. Previous investigations provided conflicting results; while lesion load and brain volume differences have often been linked to fatigue,^11,12,63^ recent findings^64^ reported no structural neuroimaging correlations with fatigue, despite the presence of neuroinflammation. This is not surprising, given the well-known and still unsolved clinical/neuroradiological paradox in MS patients^65^; we also found no significant differences between MS-F and MS-NF groups in terms of lesion load and brain volumes. At first glance, this suggests that central fatigue, at least in MS patients with low disability scores, is mainly linked to functional alterations rather than structural/anatomical damage. However, more subtle structural abnormalities emerged from our DTI analysis, even in the absence of overt clinical manifestations at neurological examination. FA was reduced in the MS-F group, particularly in bundles of fibres associated with cognitive and motor functions, such as the cingulum, superior longitudinal fasciculus and corpus callosum.^14,66^ This reduction suggests possible impaired neural communication (and perhaps efficiency), which could lead to increased energy demand for maintaining physiological performances, contributing to central fatigue. In other words, myelin alterations in cognitive and motor networks might have triggered compensatory mechanisms that have been picked up by functional neuroimaging and neurophysiological investigations (see later in the discussion). Indeed, we also observed an increase in rsFC between nodes of the DMN, between the left temporal node and the right prefrontal node of the default mode network (Fig. 2A). This result is supported, for example, by a previous study,^67^ where authors observed higher DMN activity in MS patients with fatigue, even with low depression scores. These functional compensatory changes were even more evident considering the following neurophysiological results.

### Neurophysiology (TMS and EEG)

TMS-based measurements of cortical excitability have also provided relevant insights into the neurophysiological underpinnings of central fatigue (Table 3 and Fig. 3A). While resting motor threshold, reflecting ion channels function, was similar across the three groups, we observed reduced ICF in the MS-F group, indicating an impaired intracortical glutamatergic function.^52,68^ This is consistent with previous findings^69^ that identified an imbalance between the glutamatergic and GABAergic systems in MS patients with fatigue. The reduced ICF in our study, which has already been linked to chronic fatigue and energy depletion,^70^ suggests hypofunction of the glutamatergic system in MS-F patients. Conversely, in MF-NF patients the intracortical glutamatergic tone was statistically significant higher than both healthy controls and MS-F patients (Table 3 and Fig. 3A): this might suggest that, until glutamatergic circuits of motor areas can compensate the decreased efficiency of networks that showed reduced myelin integrity at FA analyses, fatigue is likely absent, while it could emerge when these mechanisms are no longer correctly functioning, possibly due to exhaustion. Data on movement-related EEG activity in these patients^81^ further support this hypothesis.

When considering the GABA-ergic neurotransmitter component two variables were investigated: SICI (a mainly GABA-A-ergic mediated index) and CSP (a mainly GABA-B-ergic mediated index). Differently from previous studies that showed increased SICI in fatigued patients,^24,71^ SICI did not show significant differences among groups. CSP showed a trend towards being shorter in MS-F patients compared to MS-NF. Although studies in the literature report an increase in CSP among MS-F patients,^23^ our result interpretation is influenced by the observation that patients were examined at rest rather than after performing a tiring task. Indeed, the literature shows that the CSP of the MS-F group is reduced at rest compared to the MS-NF group.^72^ It remains to be determined whether such changes are contributing to fatigue generation or are an epiphenomenon of ongoing fatigue, probably on a compensatory basis. Interestingly, administration of amantadine to counteract fatigue prolongs the CSP in fatigued muscles.^72^

Our results could be compared with recent TMS-EEG evidence showing that motor fatigue in MS is linked to abnormal M1 output and altered sensorimotor network (SMN) modulation, characterized by increased post-fatigue cortical propagation, reduced task-related TEP modulation,^73^ and greater central and supraspinal fatigue, particularly during the wearing-off phase of natalizumab.^74^

Neurophysiological measurements, including resting EEG, revealed notable differences in brain oscillatory activity, with the θ frequency band being the most sensitive to fatigue (Fig. 3B). Specifically, the MS-F group exhibited higher θ-band power in frontal regions during the eyes-open condition, and this result is well supported by previous work linking cognitive effort and cognitive fatigue to the theta band (as mentioned in the introduction section). This pattern indicates increased cognitive effort, possibly related to reduced myelin integrity within cognitive and motor networks, and challenges in inhibitory control.^75,76^ Our further EEG analyses will focus on network-level dynamics, investigating whether the symptom under study exhibits patterns similar to those reported in previous work, in which regression analyses showed that cognitive fatigue was predicted by an increased delta-band microstate associated with the visual network and a decreased beta-band microstate associated with the salience network, whereas physical fatigue was associated with a reduced beta-band microstate related to the salience network.^77^

MADRS scores were also included as covariates in the EEG analysis, which focused on resting-state power spectra. While certain EEG features, such as increased frontal and parietal α and β power, have previously been associated with depression, our initial analysis revealed that increased frontal θ power is specifically related to fatigue. However, when MADRS scores were included as a covariate, these differences were no longer significant. This suggests that the observed EEG effects may reflect underlying depression severity to some extent. Therefore, the MADRS scale appears to be an important factor influencing EEG measures and should be carefully considered in future research investigating the neural correlates of fatigue.^78^

### Correlations and classification

Before applying the decision tree classification model, we first examined which key parameters correlated with the FSS values. Significant correlations were found with the ICF, all FA values, and rs-fMRI measures (Table 3). Regarding the EEG data, we focused solely on θ-band electrode power during the EO condition, based on consistent findings in the literature; however, this measure did not correlate with scores on the FSS; other EEG bands were not considered in the decision classification model because of their lack of significant discriminative meaning between MS-F and MS-NF. Ultimately, by combining all significantly correlated variables with the FSS score, we computed the overall accuracy (89.5%) for group assignment (Fig. 4). Our classification analysis identified both ICF and significantly lower FA values as the most discriminative factors between the MS-F and MS-NF groups, with an accuracy of 84.2%. This suggests that not only subtle structural damage related to myelin sheath integrity but also reduced glutamatergic activity could be key factors underlying fatigue. This would challenge previous studies that have linked the glutamatergic system only to inflammation,^79^ whose markers were instead within physiological limits in our study.

### Limitations of the study

A limitation of the study is the relatively small sample size, which, although comparable to or larger than those of previous studies on the topic, may limit the generalizability of the findings. However, the sample was highly homogeneous in terms of demographic, clinical and treatment features, and sufficiently powered for the specific aims of the study. Higher depression levels in the MS-F group might represent an interpretative confounding of results. However, MS-F patients showed very mild to mild levels of depression (not requiring pharmacological treatment), and MADRS scores were considered covariates in statistical analyses. The study is monocentric, but it should be regarded as a preliminary report on a larger 3-year project, and the methodology could be replicated in other MS centres that have access to neurophysiology and neuroimaging procedures. We will be able to conduct a longitudinal study thanks to these preliminary results. This will allow us to observe any changes in the studied parameters over time. It will also allow us to consider treatments to reduce symptom fatigue in these patients. These treatments will be based on non-invasive brain stimulation protocols. These protocols will be based on results from previous studies.^77^

## Conclusion

This study highlights the utility of a multimodal approach in uncovering a distinctive ‘fingerprint’ of MS central fatigue. The findings suggest that reduced myelin sheath integrity, even if subtle enough to remain clinically silent, might trigger a cascade of functional changes that are initially adaptive for compensation in non-fatigued patients and then become dysfunctional, sustaining the symptom.

These insights might inform not only the pathophysiology of fatigue but even future therapeutic interventions. Employing rapid diagnostic tools, such as TMS for ICF estimation and resting state EEG protocols, could potentially be implemented in a clinical setting for. fatigue assessment, expanding the significance of subjective scales. Additionally, new therapeutic approaches, which include non-invasive brain stimulation techniques based on different forms of transcranial electrical or magnetic stimulation,^80,82^ could be better targeted by these functional loco-regional changes. Among these neuromodulatory treatments, transcranial random noise stimulation has already been successful in reducing cognitive fatigue in healthy individuals engaged in demanding truck-driving tasks.^83^

## Supplementary Material

fcag134_Supplementary_Data

## Data Availability

Due to privacy and consent restrictions, individual-level neuroimaging, neurophysiological, clinical and behavioural data are not publicly available but can be shared in anonymized form upon request to the corresponding author, subject to institutional approvals. No new MATLAB codes were generated.
