# Supplementary material for: Neurophysiological, imaging and neurobiological markers of central fatigue in multiple sclerosis
Source: Brain Commun. 2026 Apr 16;8(3):fcag134. doi: 10.1093/braincomms/fcag134 (PMC13148770; doi:10.1093/braincomms/fcag134)
Supplement: fcag134_Supplementary_Data [file fcag134_supplementary_data.docx]

**Neurophysiological, imaging, and neurobiological markers of central fatigue in multiple sclerosis**

Alberto Benelli^1†^, Elisa Tatti^2†^, Rosa Cortese^3^, Elisa Massucco^1^, Ludovico Luchetti^3,4^, Marco Battaglini^3,4^, Javier Cudeiro^5^, Anna de Mauro^3^, Jian Zhang^6^, Domenico Plantone^3^, Patrizio Pasqualetti^7^, Delia Righi^3^, Francesco Neri^1,8^, Maria Laura Stromillo^3^, Alessandra Cinti^1^, Alessandro Giannotta^1^, Francesco Lomi^1^, Adriano Scoccia^1^, Giuseppe Lai^9^, Nicola De Stefano^3^, Monica Ulivelli^3^, Simone Rossi^1,3,8^

**Author affiliations:**

^1^ Siena Brain Investigation & Neuromodulation Lab (Si-BIN Lab), Department of Medicine, Surgery and Neuroscience, University of Siena, Siena, Italy

^2^ Department of Molecular, Cellular & Biomedical Sciences, City University of New York, School of Medicine, New York, NY, United States

^3^ UOC Neurologia, Department of Medicine, Surgery and Neuroscience, University of Siena, Siena, Italy

^4^ Siena Imaging SRL, Siena, Italy

^5^ Department of Physiotherapy, Medicine, and Biomedical Sciences, NEUROcom (Neuroscience and Motor Control Group), CICA (Interdisciplinary Center for Chemistry and Biology), and Galician Brain Stimulation Centre, Universidade da Coruña, A Coruña, Spain

^6^ The First Affiliated Hospital, Guangxi Medical University, Nanning, China

^7^ Health Statistics, University La Sapienza, Roma, Italy

^8^ Oto-Neuro-Tech Conjoined Lab, Policlinico Le Scotte, University of Siena, Siena, Italy

^9^ Goldsmiths, UK Department of Psychology, University of London, London, UK

^†^**Alberto Benelli and Elisa Tatti contributed equally to this work.**

**Correspondence to:** Alberto Benelli

Siena Brain Investigation & Neuromodulation Lab (Si-BIN Lab), Department of Medicine, Surgery and Neuroscience, University of Siena, Italy

Email: albertobenelli21@gmail.com

**Running title**: Central Fatigue in MS: A Multimodal Study

**Key words:** Multiple Sclerosis; Central Fatigue; Neurophysiology; Structural connectivity; Functional connectivity

**Supplementary Material**

**Neuropsychological and Clinical Assessment**

Analyses performed on cognitive test scores from the Rao Brief Repeatable Battery reported no significant differences among groups (Supplementary Table 1).

**Blood samples**

No differences were observed concerning the blood sample metrics between the two groups of patients. The blood samples were used to evaluate the concentrations of NfL and GFAP, as well as the levels of IL-4, IL-2, CXCL10 (IP-10), IL-1β, TNF-α, CCL2 (MCP-1), IL-17A, IL-6, IL-10, IFN-γ, IL-12p70, CXCL8 (IL-8), and TGF-β1. Supplemental Table 3 reports all p-values group comparisons.

**EEG**

When MADRS is used as a covariate, the differences in theta-band power during the open-eye condition are cancelled out (Supplementary Table 4).

**Neuroimaging**

**Brain Volumetric Assessment**

Comparing cortical and subcortical volumes between the groups, the only differences that have been observed concern the caudate nucleus and the thalamus (TH). Relative to the caudate nucleus, the analyses showed an effect of the group variable (F**_(2)_**= 5.607, p= .006, η2= 0.162), suggesting a moderate effect size. Post-hoc tests with Bonferroni corrections revealed a significantly lower volume related to MS-F compared to HC (Mean Difference = -1.118, p = .004). For the thalamus volume data, results showed a highly significant main effect of the group (F=12.116, p<0.001, η2=0.293), reflecting a large effect size (Supplementary Figure 2).

**GM atrophy and WM lesion distributions**

The permutation test did not show any differences in the GM volumes of the two groups of MS patients.

MS patients, both with fatigue (Supplementary Figure 2A) and without fatigue (Supplementary Figure 2B), showed a significant difference in GM volumes compared to HC. As shown by the image below, MS-F showed significantly greater atrophy compared to HC in the TH region. Finally, MS patients, both with fatigue (Supplementary Figure 2C) and without fatigue (Supplementary Figure 2D), showed a significantly higher lesion load compared to HC. However, no differences in the lesion load were observed concerning the number of lesions and lesion volume between the two groups of MS patients.

**Supplementary Figures**

**Supplementary Figure 1 | MADRS group comparisons:** MADRS comparisons between groups. The central line represents the median, while the whiskers at the ends correspond to the minimum and maximum values. Data from 53 subjects were used for both analyses. For the MADRS scores analysis the Kruskal-Wallis H test has been used. **** p < .0001. Most MS F patients showed a very mild-to-mild degree of depression.

**
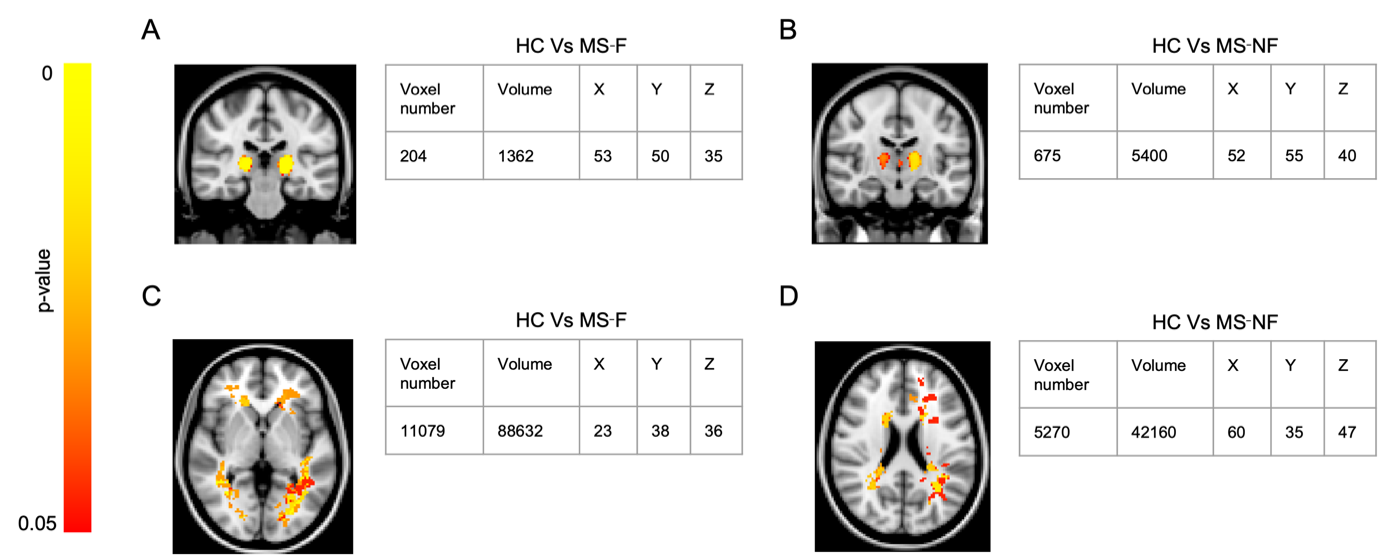
**

**Supplementary Figure 2 | Brain volume group comparisons:** Panels **A** and **B** show the coronal representation of the GM atrophy localized in the Thalamus region, comparing respectively MS-F with HC and MS-NF with HC; tables on the right of the coronal scans represent the X, Y, Z coordinates, and volume size of the most significant voxel in MNI space. Panels **C** and **D** show the axial representation of the LPM, comparing respectively MS-F with HC and MS-NF with HC; tables on the right of the coronal scans represent the X, Y, Z coordinates, and volume size of the most significant voxel in MNI space. Data from 62 subjects were used for both analyses. For both the GM atrophy and LPM analyses the ANCOVA test has been used.

**Supplementary Tables**

**Supplementary Table 1** | *MS patient therapies*

| **Active principle** | Total | MS-F | MS-NF |
| --- | --- | --- | --- |
| Dimethyl fumarate | 18 | 7 | 11 |
| Natalizumab | 4 | 3 | 1 |
| Pregabalin | 2 | 1 | 1 |
| Interferon | 1 | 0 | 1 |
| Cladribine | 3 | 0 | 3 |
| Ocrelizumab | 2 | 2 | 0 |
| Gabapentin | 2 | 2 | 0 |
| Fingolimod | 1 | 0 | 1 |
| Fluticasone furoate | 1 | 0 | 1 |
| Ofatumumab | 1 | 1 | 0 |
| Omalizumab | 1 | 1 | 0 |

**Supplementary Table 2** | *p-values,* BRB-NT score group comparisons

|  | **MS-F Vs MS-NF** | **MS-F Vs HC** | **MS-NF Vs HC** |
| --- | --- | --- | --- |
| **SRT-LTS** | .53 | 1.00 | 1.00 |
| **SRT-CLTR** | .64 | 1.00 | 1.00 |
| **SPART** | 1.00 | 1.00 | 1.00 |
| **SDMT** | 1.00 | 1.00 | 1.00 |
| **PASAT 3** | 1.00 | 1.00 | .84 |
| **PASAT 2** | 1.00 | .46 | 1.00 |
| **SRT-D** | 1.00 | 1.00 | 1.00 |
| **SPART-D** | 1.00 | 1.00 | 1.00 |
| **WLG** | .13 | .20 | 1.00 |

***Note:*** *SRT-LTS = Selective Reminding Test- Long Term Storage; SRT-CLTR = Selective Reminding Test- Consistent Long-Term Retrieval; SPART = Spatial Recall Test; SDMT = Symbol Digit Modalities Test; PASAT = Paced Auditory Serial Addition Test; SRT-D = Selective Reminding Test- Delayed; SPART-D = Spatial Recall Test - Delayed; WLG = Word List Generation test*

**Supplementary Table 3** | *p-values* for blood sample group comparisons

|  | **MS-F Vs MS-NF** | **MS-F Vs HC** | **MS-NF Vs HC** |
| --- | --- | --- | --- |
| **NfL** | 1.00 | .08 | .09 |
| **GFAP** | 1.00 | 1.00 | 1.00 |
| **IL-4 (A4)** | .58 | .064 | .61 |
| **IL-2 (A5)** | 1.00 | .11 | .41 |
| **IP-10 (A6)** | .90 | .42 | 1.00 |
| **IL-1β (A7)** | .46 | .16 | 1.00 |
| **TNF-α (A8)** | .55 | .20 | 1.00 |
| **MCP-1 (A10)** | 1.00 | .87 | .93 |
| **IL-17A (B2)** | 1.00 | .35 | .72 |
| **IL-6 (B3)** | 1.00 | .16 | .21 |
| **IL-10 (B4)** | .50 | .01 | .20 |
| **IFN-γ (B5)** | .71 | .26 | 1.00 |
| **IL-12p70 (B6)** | 1.00 | .007 | .029 |
| **IL-8 (B7)** | .254 | .08 | 1.00 |
| **TGF-β1 (Free Active) (B9)** | 1.00 | .72 | 1.00 |

**Supplementary Table 4** | *p-values,* Theta Band EEG group comparisons using MADRS as covariate

|  | **MS-F Vs MS-NF** | **MS-F Vs HC** | **MS-NF Vs HC** |
| --- | --- | --- | --- |
| θ **FC1** | .786 | .630 | .161 |
| θ **F1** | 1.00 | .512 | .105 |
| θ **F3** | 1.00 | 1.00 | .093 |
